# TAMeless traitors: macrophages in cancer progression and metastasis

**DOI:** 10.1038/bjc.2017.356

**Published:** 2017-10-24

**Authors:** Shweta Aras, M Raza Zaidi

**Affiliations:** 1 Fels Institute for Cancer Research and Molecular Biology, and Department of Medical Genetics and Molecular Biochemistry, Lewis Katz School of Medicine at Temple University, Philadelphia, PA 19140, USA

**Keywords:** tumour-associated macrophages, spectrum polarisation, cancer, invasion, metastasis, immunosuppression

## Abstract

Macrophages are conventionally classified into M1 and M2 subtypes according to their differentiation status and functional role in the immune system. However, accumulating evidence suggests that this binary classification system is insufficient to account for the remarkable plasticity of macrophages that gives rise to an immense diversity of subtypes. This diverse spectrum of macrophage subtypes play critical roles in various homeostatic and immune functions, but remain far from being fully characterised. In addition to their roles in normal physiological conditions, macrophages also play crucial roles in disease conditions such as cancer. In this review, we discuss the roles tumour-associated macrophages (TAMs) play in regulating different steps of tumour progression and metastasis, and the opportunities to target them in the quest for cancer prevention and treatment.

Macrophages constitute a prominent set of immune cells that are phagocytic in nature and are present in almost all tissues. In general, they are differentiated cells of mononuclear origin and display specific phenotypic characteristics. In mice, macrophages show surface expression of markers such as CD11b, F4/80 and colony-stimulating factor-1 receptor (CSF-1R) and do not express Gr1; whereas, in humans, macrophages show expression of CD68, CD163, CD16, CD312 and CD115 (Qian and Pollard, 2010).

Macrophages are an incredibly diverse set of cells, constantly altering their functional state in response to environmental stimuli. They undergo the ‘polarisation’ process wherein they express different surface markers and functional programs in response to microenvironmental stimuli such as the cytokines and other signalling mediators. According to the binary polarisation concept, there exist two polarisation states of macrophages: Classically activated macrophages (M1) produce pro-inflammatory cytokines and reactive oxygen/nitrogen species, which are crucial for host defence and tumour cell killing and, therefore, are considered as ‘good’ macrophages. The alternatively activated macrophages (M2) produce anti-inflammatory cytokines and are involved in the resolution of inflammation. These are considered ‘bad’ macrophages because they not only suppress the destructive immunity against parasites and tumour cells, but also promote angiogenesis and matrix remodelling, which make the tumour microenvironment conducive to tumour progression and metastasis (Huang and Feng, 2013).

Macrophages not only perform vital functions in normal physiological conditions such as development, wound healing, infection and maintenance of tissue homeostasis, but are also involved in a variety of disease conditions such as autoimmune disorders, atherosclerosis and tumourigenesis (Wynn *et al*, 2013). Tumourigenesis is a highly complex, multi-step process and many findings provide strong evidence for the role of specific subsets of macrophages in tumourigenesis. These macrophages are commonly referred to as ‘tumour-associated macrophages (TAMs)’. Tumour-associated macrophages were thought to closely resemble the M2 phenotype; however, findings discussed in this review suggest that this binary polarisation model is going obsolete, and there exists a whole spectrum of TAM phenotypes that are yet to be discovered and fully characterised. Tumour-associated macrophages also contribute to many steps of tumourigenesis, such as transformation, tumour cell proliferation, angiogenesis, invasion and metastasis. In this review, we summarise various physiological and functional aspects of TAMs, as well as their roles in regulating various steps of tumour initiation, progression and metastasis. This review will help shed light on the potential of TAMs as prognostic biomarkers for various cancers, as well as ways to target them for therapeutic interventions.

## The two faces of macrophages

The role of macrophages in tumourigenesis has been controversial. Macrophages have conventionally been considered to be anti-tumourigenic in nature, play tumour-suppressive roles, and illustrate a significant link between high density and better prognosis, particularly in the case of colorectal cancer. For instance, Ong *et al* showed that macrophages in a colorectal cancer model are pro-inflammatory, and inhibited the growth of tumour cells by secreting chemokines to attract T cells, thereby priming an anti-tumour type-1 inflammatory response (Ong *et al*, 2012). It was also reported that when stimulated with TLR ligands, anti-CD40 or interferon-γ (IFN-γ), TAMs show anti-tumoural functions, provided CD47 expression on cancer cells does not inhibit tumour cell phagocytosis (Jaiswal *et al*, 2009). Moreover, pro-inflammatory macrophages were also shown to have tumour suppressive effects via production of reactive oxygen species and reactive nitrogen intermediates (Vicetti Miguel *et al*, 2010). However, many subsequent studies challenged this notion and indicated that macrophages also display pro-tumourigenic properties. In a mouse model of breast cancer, Lin *et al* showed that a homozygous null mutation of a gene encoding the macrophage growth factor, colony stimulating factor-1 (CSF-1), not only reduced macrophage infiltration but also completely abolished tumour progression and metastasis. On the contrary, overexpression of this CSF-1 protein increased the rate of tumour progression and metastasis (Lin *et al*, 2001). Moreover, inhibition of the CSF-1 receptor signalling pathway by virtue of a small molecule inhibitor abrogated infiltration of TAMs and enhanced recruitment of CD8+ T cells, thereby reducing cervical and mammary tumour growth (Strachan *et al*, 2013). According to Shree *et al*, cathepsin-expressing macrophages protected against chemotherapy-induced tumour cell death in breast cancer, and cathepsin inhibition significantly reversed this phenomenon (Shree *et al*, 2011). Recently, Gordon *et al*, showed that both mouse and human TAMs express programmed cell death protein 1 (PD-1), thereby negatively regulating their phagocytic activity against tumour cells. Blockade of this PD-1/PD-L1 axis restores phagocytic activity by these TAMs, reduces tumour growth and lengthens survival of mice, strongly suggesting a pro-tumourigenic potential of these TAMs (Gordon *et al*, 2017).

Macrophages are multifaceted and highly plastic in their characteristics. The classically activated M1 macrophages are stimulated by microbial substrates such as lipopolysaccharide, toll-like receptor ligands and cytokines such as IFN-γ, and are involved in Th1 type of responses. Once activated, M1 macrophages are characterised by secretion of pro-inflammatory cytokines such as interleukins IL6, IL12, IL23 and tumour necrosis factor-α, and have a strong microbicidal and tumouricidal functions. Phenotypically, they express high levels of major histocompatibility complex class II (MHC-II), CD68, and CD80 and CD86 costimulatory molecules.

**Figure 1:**
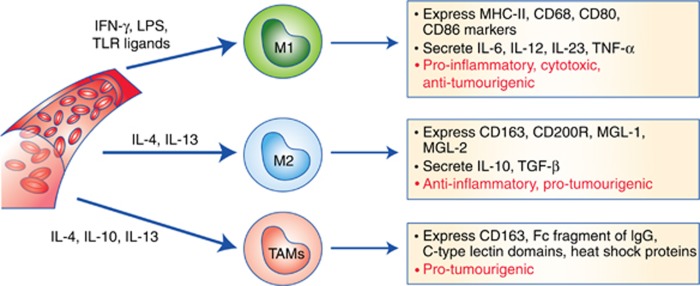
**Macrophage differentiation and their role in tumourigenesis.** Classically activated (M1) macrophages are activated by IFN-γ, LPS or TLR ligands, secrete pro-inflammatory cytokines and play tumouricidal roles. Alternatively activated (M2) macrophages are activated by IL-4 and IL-13, secrete anti-inflammatory cytokines IL-10 and TGF-β and play tumourigenic roles. Tumour-associated macrophages (TAMs) display M2-like phenotype and exhibit pro-tumourigenic features.

The alternatively activated M2 macrophages are stimulated by IL4 and IL13, secrete IL10, transforming growth factor-β (TGF-β), and chemokines, and are involved in tissue remodelling and tumour progression (Fan *et al*, 2016). Phenotypically, M2 macrophages express low levels of MHC-II and feature expression of CD163 (Barros *et al*, 2013), CD200R membrane glycoprotein (Jaguin *et al*, 2013) as well as high levels of MGL1 and MGL2, which are members of the macrophage galactose type C-lectin family (Raes *et al*, 2005). A genetic profile for M2 macrophages showed upregulation of the genes arginase 1 (*Arg1*), MMR (*Mrc1*), resistin-like molecule α (*FIZZ1*), and chitinase-like protein *Ym1* (Raes *et al*, 2002). Tumour-associated macrophages in the tumour microenvironment exhibit M2-like polarisation state of macrophages with pro-tumourigenic functions because they express a series of markers, such as CD163, the Fc fragment of IgG, C-type lectin domains, and heat shock proteins, some of which are commonly expressed in M2-macrophages. Moreover, acquisition of an M2-like phenotype is also caused by secretion of tumour-derived cytokines such as IL4, IL10, and IL13 (Sica *et al*, 2002; Sakai *et al*, 2008) (Figure 1).

## The classification conundrum

A large body of research clearly suggests that the historical binary classification of macrophages is grossly oversimplified, and represents two extremes of their activation states. In view of some recent findings about macrophage activation, the classical M1/M2 polarisation model seems to be obsolete as it fails to fully account for the complexity of the macrophage activation process. Xue *et al* recently showed that by virtue of highly specific and standardised stimulation of human macrophages, the current M1/M2 paradigm can be expanded into a ‘spectrum model’ (Xue *et al*, 2014). This model suggests that due to the presence of a network of transcriptional regulators, there exists a spectrum of differentiated macrophages, many of which are yet to be fully discovered.

A recently published report identified a few other categories of macrophages with molecular phenotypes that do not fit the conventional M1 or M2 types, but have been involved as main players in various human pathologies. For example, the antigen CD169 (Siglec-1) is highly expressed on and is reported as a marker of one macrophage subpopulation found in bone marrow, lymph node, liver, and spleen. Although the information about the signalling pathway involved in the activation of CD169+ macrophages is imprecise, CD169+ macrophages are mainly involved in erythropoiesis and immune regulation (Chow *et al*, 2013). Another non-M1/M2 subtype of macrophages expresses T-cell receptor (TCR). T-cell receptor is required for antigen recognition, and several reports have suggested the presence of murine and human TCR+ macrophages, especially TCRαβ^+^ and TCRγδ^+^ macrophages, in inflammatory and infectious diseases (Chavez-Galan *et al*, 2015). Georgoudaki *et al* recently identified a novel subtype of TAMs with an M2-like immunosuppressive gene profile expressing a novel receptor ‘macrophage receptor with collagenous structure’ or ‘MARCO’ in mouse tumour models of mammary carcinoma, colon cancer and B16 melanoma (Georgoudaki *et al*, 2016).

**Figure 2:**
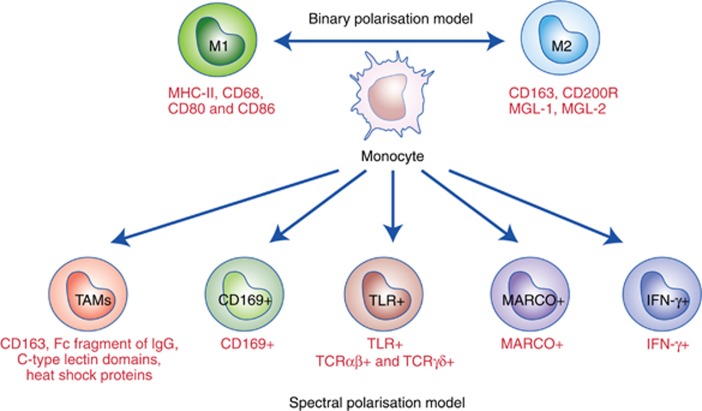
**The ‘binary’ *vs* ‘spectrum’ model of macrophage polarisation.** Recent evidence strongly suggests that the conventional model of binary polarisation of macrophages into M1 and M2 subtypes is oversimplified and the molecular profile of several newly discovered subtypes of macrophages do not fit either phenotype. The spectrum model of macrophage polarisation suggests that there exist various subtypes of differentiated macrophages by virtue of an intricate network of transcriptional regulators, which participate in many homeostatic as well as pathological functions.

Another example of non-M1/M2-type macrophages is IFN-γ-secreting macrophages. Interferon-γ forms an important constituent of the innate immune defence system and secretion of IFN-γ by human NK cells and T cells in response to interleukins has been long established. However, IFN-γ secretion by macrophages is still controversial owing to the doubts about contamination of macrophages by NK or T cells. However, a growing body of evidence suggests that macrophages are capable of secreting IFN-γ both *in vitro* and *in vivo*. For instance, Darwich *et al* showed that at single cell level, human macrophages secrete IFN-γ after induction with interleukins IL-12 and IL-18 (Darwich *et al*, 2009). Robinson *et al* showed a similar phenomenon of induction of IFN-γ secretion post *Mycobacterium tuberculosis* infection of human macrophages (Robinson *et al*, 2010). We have also identified macrophages secreting IFN-γ, which promoted melanoma growth in an allograft mouse model (Zaidi *et al*, 2011) (Figure 2).

Given the importance of macrophages in homeostatic and pathological conditions, a thorough investigation of the multiple factors in normal and diseased microenvironments is absolutely warranted to dissect the mechanisms of macrophage activation, plasticity, and polarisation.

## TAMs in tumour microenvironment

Tumour-associated macrophages originate from the circulating peripheral blood monocytes, which are derived from the bone marrow. These monocytes are recruited to the tumour tissues and then differentiate locally in response to a variety of cytokines, chemokines, and growth factors produced by the stromal and tumour cells in the tumour microenvironment. For instance, the chemokine CCL2 and macrophage colony-stimulating factor were shown to recruit inflammatory monocytes to the tumour site, and then differentiate into TAMs in response to IL-4, IL-10, IL-13 and other cytokines in the tumour microenvironment and promote tumour metastasis (Qian *et al*, 2011). Another report suggested that hypoxia-inducible chemotactic factors such as the CXCR4 ligand CXCL12 and Angiopoietin-2 (Ang-2) promote recruitment of Tie2-expressing monocytes in hypoxic areas of tumours and differentiate them into Tie2-expressing macrophages (Murdoch *et al*, 2007). Some other microenvironmental factors such as CSF-1, CCL2, IL6, vascular endothelial growth factor (VEGF-A) and platelet-derived growth factor (PDGF) have also been involved in infiltration of monocytes to the tumour sites (Balkwill, 2004; Joyce and Pollard, 2009).

**Figure 3:**
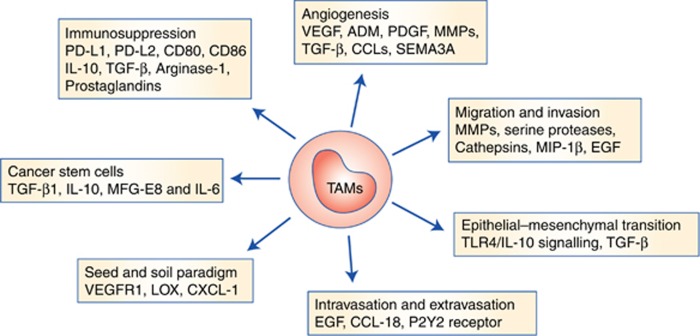
**Role of TAMs in tumourigenesis.** Different roles of TAMs in promoting tumour invasion and metastasis, along with the specific markers, are depicted. Tumour microenvironmental cues educate the macrophages to adopt a specific phenotype and perform distinct roles contributing towards tumourigenesis.

It is now well-established that the majority of malignant tumours contain macrophages as a major component of their tumour microenvironment. Upon stimulation, these TAMs secrete a wide variety of cytokines, growth factors, inflammatory substrates and proteolytic enzymes that play major roles in cancer progression (Figure 3). Moreover, clinicopathological studies have shown that there also exists a strong correlation between increased macrophage density and poor prognosis in lung, hepatocellular carcinoma, renal cell carcinoma (Komohara *et al*, 2011) and breast cancer (Campbell *et al*, 2011; Mahmoud *et al*, 2012; Medrek *et al*, 2012). Moreover, according to a recent report by Wang *et al*, macrophages were also shown to play a critical role in melanoma resistance to BRAF inhibitors (Wang *et al*, 2015). Therefore, TAM infiltration can potentially be used as a prognostic marker of clinical outcomes for many cancers and can be potentially targeted for cancer prevention or treatment.

The leading or invasive edge of the primary tumour is a crucial site in the tumour microenvironment where immune cells and stromal cells are recruited and play immunosuppressive roles. TAMs are located at the perivascular areas or at the invasive edge of the tumours and are recruited there by tumour-derived chemoattractants. Upon arrival, TAMs supply pro-migratory factors such as epidermal growth factor (EGF), promote proteolytic remodelling of the extracellular matrix, accelerate tumour motility and induce migration and invasion of tumour cells (Quail and Joyce, 2013). According to a report by Wyckoff *et al* (2007), multiphoton microscopy of mouse mammary tumours showed large number of macrophages at the margins of the tumours. Apart from the perivascular region of the tumour where they promote tumour cell invasion, TAMs are also reported to get recruited in the hypoxic regions of the tumour (Wyckoff *et al*, 2004).

The tumour microenvironment is a complex ecology of heterogeneous cell populations, which have a robust influence on tumourigenesis. For example, TAM–adipocyte interactions have been shown to drive cancer initiation and progression in case of obesity and overweight conditions. Adipocyte hypertrophy, inflammation and apoptosis results in macrophage recruitment, which phagocytose dead/dying adipocytes and develop inflammatory foci called crown-like structures (CLS). Many reports have shown that these adipocytes and apoptotic CLSs promote tumour progression in breast (Morris *et al*, 2011) and ovarian cancers (Nieman *et al*, 2011). Apart from TAMs, cancer-associated fibroblasts (CAFs) constitute another main component of infiltrating stromal cells that are reported to be involved in tumour progression (Komohara and Takeya, 2017). Recently, Hashimoto *et al* (2016) showed that cell–cell interaction between TAMs and CAFs promoted recruitment and activation of each other and contributed to neuroblastoma progression. Similarly, Miyake *et al* (2016) showed that high CXCL1 levels in urothelial cancer of the bladder cells resulted in enhanced recruitment of TAMs/CAFs, higher metastatic potential, and poor prognosis. In another report, CAFs were shown to promote an immunosuppressive microenvironment through the induction and accumulation of pro-tumoural macrophages, suggesting a strong crosstalk between microenvironmental stromal cells (Takahashi *et al*, 2017).

## TAMs in angiogenesis

In many cancers, benign-to-malignant transition is associated with a significant increase in vascularisation, a process known as angiogenesis, which provides cancer cells nutrients and oxygen to allow them to multiply, invade and metastasise. This process of forming new vasculature is highly complex and TAMs are one of the major contributors in this process (Lin and Pollard, 2007). According to a recent report, quantitative analysis and assessment of the spatial associations between TAMs and tumour neovasculature demonstrated the great significance and close association of TAMs and tumour angiogenesis during cervical cancer development and progression (Jiang *et al*, 2016). Neovascularisation is induced when TAMs secrete pro-angiogenic factors, such as VEGF, adrenomedullin (ADM), PDGF, TGF-β and matrix metalloproteinases (MMPs). For instance, VEGF-A was reported to contribute to neoangiogenesis and macrophage recruitment at the tumour site in a mouse model of skin carcinogenesis (Linde *et al*, 2012). Moreover, TAMs were shown to sense hypoxia in avascular areas within tumours and release VEGF-A, a very potent pro-angiogenic factor (Laoui *et al*, 2014). Another report on Merkel cell carcinoma, a highly malignant neuroendocrine tumour of the skin, shows that TAMs express high levels of VEGF-C, which promotes lymphovascularisation (Werchau *et al*, 2012; Matsumoto-Okazaki *et al*, 2015). These reports strongly demonstrate the role of VEGF-producing TAMs in angiogenesis and tumour progression. Chen and colleagues found that infiltrating TAMs produced ADM when co-cultured with melanoma cells. There was also a significant improvement in endothelial cell proliferation and tube formation with ADM and this effect was abrogated upon administration of neutralising ADM antibody *in vitro*, suggesting the pro-angiogenic action of TAM-derived ADM (Chen *et al*, 2011b).

Apart from VEGFs and ADM, MMPs were also shown to be expressed by TAMs and involved in angiogenesis. According to a recent report, MMP9 secretion by TAMs recruited into the tumour site, in response to osteopontin signalling in melanoma, induced angiogenesis and tumour growth. These reports suggest that MMP9 aids tumour progression by remodelling the extracellular matrix and by promoting neoangiogenesis (Kale *et al*, 2015). Macrophage differentiation and chemotaxis is regulated by growth factors such as CSF1. Studies involving TAM depletion in breast tumours using *Csf1*-null mutation displayed substantial reduction in angiogenic potential and tumour burden, suggesting that these macrophages are required for angiogenesis. Additionally, this TAM depletion was reversed upon rescuing CSF1 in breast epithelium. Furthermore, overexpression of CSF1 in wild-type mice led to premature accumulation of macrophages in lesions and a dramatic increase in angiogenesis. It has also been demonstrated that most TAM depletion strategies using liposome-encapsulated clodronate inhibits angiogenesis in tumour models (Gazzaniga *et al*, 2007; Halin *et al*, 2009).

Hypoxia is a major contributor in angiogenesis. Dual staining of hypoxia and macrophage markers reveals massive infiltration of TAMs in hypoxic/necrotic regions of the tumour. This massive recruitment of TAMs is usually facilitated by chemokines like CCL2, CCL5, VEGF, CSF-1, semaphorin 3A (SEMA3A), endothelin, eotaxin and oncostatin M. Once macrophages arrive in these tumour compartments, their migration is halted via hypoxia-dependent mechanisms. Pro-tumoural functions of macrophages are then facilitated by a hypoxia-dependent transcription factor HIF1a, which induces expression of a large set of genes associated with angiogenesis such as VEGF (Henze and Mazzone, 2016). This pro-tumoural function of hypoxic TAMs was validated by Casazza *et al*, wherein they showed that macrophage-specific genetic deletion of Nrp-1, a binding partner of hypoxia induced TAM attractant Semaphorin 3A (Sema3A), prevented macrophage entry into the hypoxic region and ablated pro-angiogenic and immunosuppressive functions of TAMs, thereby inhibiting tumour growth and metastasis (Casazza *et al*, 2013).

## TAMs in migration and invasion

The potential of tumour cells to invade and metastasise depends on the tumour microenvironment. Since TAMs constitute a major component of the tumour microenvironment, they play a crucial role in facilitating these processes. TAMs primarily promote tumour cell invasion and metastasis via secretion of matrix metalloproteinases, serine proteases, and cathepsins, which alter the composition of the ECM by modifying cell–cell junctions and promoting basal membrane disruption. For instance, high levels of cathepsin protease activity are induced in the majority of macrophages in the microenvironment of pancreatic islet cancers, mammary tumours, and lung metastases during malignant progression. Furthermore, TAM-secreted cathepsins B and S were critical for promoting pancreatic tumour growth, angiogenesis, and invasion *in vivo*, and also markedly enhanced the invasiveness of cancer cells in culture (Gocheva *et al*, 2010). According to a recent report, STAT3 and STAT6 were shown to synergistically promote cathepsin secretion by macrophages, thereby enhancing tumour invasion and metastasis. Genetic deletion of *Stat3* and *Stat6* impaired tumour development and invasion *in vivo*. Together, these findings demonstrate that STAT3 and STAT6 cooperate in macrophages and enhance tumour progression in a cathepsin-dependent manner (Yan *et al*, 2016).

Recently, Baghel and colleagues showed that a macrophage-derived protein MIP-1β potentiated cancer cell invasion and metastasis via upregulation of MYO3A gene within breast cancer cells. Moreover, there was also a significant correlation between higher expression of this protein and poor survival of breast cancer patients, thereby validating the findings (Baghel *et al*, 2016). A novel real-time multiphoton imaging system developed by Wyckoff *et al* to investigate the metastatic nature of tumour cells demonstrated that invasion of breast cancer cells occurred in association with TAMs in mammary tumours, which is in agreement with the notion that TAMs support tumour invasion and metastasis (Wyckoff *et al*, 2007). In another clinicopathological study on breast carcinoma by Yang *et al*, the infiltration densities of TAMs were significantly higher in breast cancer patient specimens as compared to adjacent normal tissue (Yang *et al*, 2015). Moreover, in pancreatic tumours, targeting TAMs by inhibiting either the myeloid cell receptors colony-stimulating factor-1 receptor (CSF1R) or chemokine (C-C motif) receptor 2 (CCR2) decreased the number of tumour-initiating cells (TIC) and inhibited metastasis (Mitchem *et al*, 2013). A more recent study demonstrated that Warburg metabolism in tumour-conditioned macrophages promoted vascularisation, augmented extravasation of tumour cells from blood vessels, and metastasis in human pancreatic ductal adenocarcinoma. Furthermore, inhibition of glycolysis in TAMs with a competitive inhibitor disrupted this metastatic phenotype, reversing the observed increases in TAM-supported angiogenesis, extravasation, and epithelial–mesenchymal transition (EMT) (Penny *et al*, 2016).

TAMs have also been reported to play crucial role in ovarian cancer growth, invasion and metastasis. Using an established mouse model for epithelial ovarian cancer, Yin *et al* showed that TAMs promote spheroid formation and tumour growth by secreting EGF. Activation of EGFR on tumour cells by EGF in turn upregulated VEGF/VEGFR signalling in surrounding tumour cells to support tumour cell proliferation and migration. Pharmacological blockade or antibody neutralisation of EGFR in TAMs abrogated spheroid formation and ovarian cancer progression in mouse models. These findings suggest that EGF secreted from TAMs plays a critical role in promoting early metastasis of ovarian cancer (Yin *et al*, 2016). Moreover, TAMs were also shown to promote invasion via toll-like receptor signalling in patients with ovarian cancer (Ke *et al*, 2016).

## TAMs in epithelial–mesenchymal transition

Epithelial–mesenchymal transition plays a critical role in tumour progression and metastasis wherein polarised epithelial cells change their phenotype to motile mesenchymal cells. Recent studies have shown that TAMs are one of the orchestrators of this process, which involves loss of cell–cell contact and acquisition of a migratory phenotype. Epithelial–mesenchymal transition is characterised by suppression of epithelial markers such as E-cadherin, and upregulation of mesenchymal markers, including Vimentin, Slug, Snail, Fibronectin, zinc-finger E-box binding homeobox 1 (ZEB1), ZEB2, and α-smooth muscle actin, as a result of which the cells acquire the ability to migrate and invade, leading to tumour progression and metastasis. Regulation of EMT is mediated by many growth factors and cytokines such as TGF-β, forkhead box protein M1 (FoxM1), hepatocyte growth factor, EGF, NFκB, Notch, and Wnt (Zhang *et al*, 2015). A growing body of evidence has shown the important contribution of TAMs in EMT. For instance, Liu *et al* showed that M2-polarised TAMs promoted EMT in pancreatic cancer cells, partially through the TLR4/IL-10 signalling pathway (Liu *et al*, 2013). According to another report, TAMs promote cancer stem cell (CSC)-like properties via TGF-β1-induced EMT in hepatocellular carcinoma (Fan *et al*, 2014).

## TAMs in intravasation and extravasation

Intravasation is the process by which tumour cells enter a local blood vessel, and it is one of the important steps in the cascade of events leading to metastasis. Macrophages have been shown to enhance the ability of cancer cells to intravasate. Multiphoton intravital imaging techniques have shown that macrophages are located at the periphery of the tumour and their density decreases towards the centre wherein they are localised to the blood vessels and assist tumour cells to intravasate into the bloodstream (Sidani *et al*, 2006; Condeelis and Weissleder, 2010). Mechanistically, tumour cells secrete CSF1, which stimulates macrophages to produce EGF that in turn activates migration of the tumour cells (Wyckoff *et al*, 2004). Epidermal growth factor and CSF1 induce formation of invadopodia in cancer cells and podosomes in TAMs, structures that degrade extracellular matrix and facilitate intravasation (Condeelis and Pollard, 2006). In case of breast cancer patients, mammary TAMs secrete CCL18 which in turn triggers integrin clustering on cancer cells. This results in adherence of these cells to extracellular matrix and promotes intravasation (Chen *et al*, 2011a).

While in circulation, platelets form aggregates with tumour cells and protect them from cytotoxic immune cell recognition. Platelets escort tumour cells in the circulation to the site of extravasation, where they help tumour cells exit the circulation into secondary organs. According to a recent study, platelets promote extravasation of tumour cells and metastatic seeding through ATP-dependent activation of the endothelial P2Y2 receptor, which opens the vessel barrier (Schumacher *et al*, 2013). Qian *et al* used an intact *ex vivo* lung imaging system and showed that tumour cells interacting with macrophages showed a higher percentage of extravasation, whereas depletion of macrophages using L-clodronate significantly reduced the number of tumour cells undergoing extravasation (Qian *et al*, 2009).

## TAMs in the seed and soil paradigm

The seed and soil hypothesis, also known as the organ tropism hypothesis, proposed a concept that before metastatic colonisation, the primary tumour secretes factors that prepares a pre-metastatic niche at a distant site to become receptive for subsequent metastasis. It is characterised by accumulation of bone marrow (BM)-derived cell types, increased numbers of fibroblasts, secreted oncoproteins, and cytokines. Kaplan *et al* showed that BM-derived VEGFR+ cells arrive at the distant pre-metastatic site well before the primary tumour cells arrive. Furthermore, depleting VEGFR1+ cells by antibody-mediated inhibition of VEGFR1 signalling or receptor mutation interfered with the formation of these pre-metastatic clusters and inhibited metastasis (Kaplan *et al*, 2005). Erler *et al* showed that the expression of copper-dependent amine oxidase called lysyl oxidase (LOX), which is secreted by hypoxic breast tumour cells and is a major target of hypoxia-inducible factor (HIF) signalling, stabilises the ECM network by cross-linking collagen IV in the basement membranes at the pre-metastatic sites. This crosslinking facilitates myeloid cell recruitment and subsequent tumour cell colonisation. Moreover, LOX ablation prevents the formation of such sites and inhibits metastatic growth (Erler *et al*, 2009). A more recent report by Wang *et al* showed that colorectal carcinoma cells secrete VEGF-A, which stimulates TAMs to produce CXCL1 in the primary tumour. Elevation of CXCL1 in premetastatic liver tissue recruited CXCR2-positive myeloid-derived suppressor cells (MDSC) to form a premetastatic niche that ultimately promoted liver metastases (Wang *et al*, 2017). Thus, TAMs play a critical role in preparing the premetastatic niche, recruitment and retention of circulating tumour cells at the metastatic site, and fostering their growth.

## Interaction with cancer stem cells

Cancer stem cells are specific subpopulations of cells within tumours that exhibit stem cell-like properties and have the potential to initiate tumours by undergoing self-renewal and differentiation. Owing to the evidence of pivotal roles of stromal cells such as TAMs, the interaction between TAMs and CSCs has become an exciting area of research. Recent studies have tried to analyse functional roles of TAMs in regulating tumour promoting activities of CSCs through complex molecular networks comprised of cytokines, chemokines and growth factors (Jinushi *et al*, 2012). Several studies have reported interaction of macrophages with CSCs in many tumour models. Yi *et al* showed a positive correlation between infiltration of macrophages and glioma initiating cancer stem cells or GICSCs. TAMs were localised in higher densities in areas with a higher number of GICSCs with close contacts between the two cell types. Moreover, in glioma tissue, the secretion of CSC-derived chemoattractants such as CCL2, CCL5, VEGF-A was much higher, facilitating recruitment of macrophages (Yi *et al*, 2011). Another report suggested that CSCs in glioma tissue induced macrophage infiltration and polarisation into M2 phenotype because these macrophages secreted a large number of cytokines, such as TGF-β1 and IL-10, and facilitated immunosuppression (Wu *et al*, 2010). Jinushi *et al* showed that TAMs interact with CSCs and promote their tumourigenic potential via production of milk fat globule-epidermal growth factor–VIII (MFG-E8) and IL-6 through coordinated activation of the STAT3 and sonic hedgehog pathways. Interestingly, CSC population is the major population promoting the production of MFG-E8 and IL-6 from macrophages, suggesting that they impart macrophages with the ability to produce tumourigenic factors such as MFG-E8 and IL-6 (Jinushi *et al*, 2011).

## TAMs in immunosuppression

The role TAMs play in immunosuppression to promote tumour progression has been widely investigated. TAMs are involved in immunosuppression either by directly inhibiting the CD8+ T-cell response through direct cell–cell interaction with T cells or by secreting immunosuppressive cytokines and proteases such as IL-10, TGF-β, Arginase-1, and prostaglandins, which inhibit T-cell activation and proliferation. For instance, macrophages express PD-L1/PD-L2 and CD80/CD86, which are the ligands of the inhibitory receptors programmed cell death protein 1 (PD-1) and cytotoxic T-lymphocyte antigen 4 (CTLA4), respectively. Activation of these receptors by their ligands results in inhibition of TCR signalling and T-cell cytotoxic function (Kuang *et al*, 2009; Ojalvo *et al*, 2009). Recently, it was shown that hypoxia also plays an important role in immunosuppression. TAMs found in hypoxic regions of the tumour upregulate PD-L1 expression via HIF-1α signalling and consequently induce T-cell suppression (Doedens *et al*, 2010; Noman *et al*, 2014). Another report suggested that, as compared to MDSCs, macrophages produced higher levels of anti-inflammatory factors and were more immunosuppressive, facilitating tumour immunoevasion in multiple murine models of breast cancers (Fang *et al*, 2017). CAFs were also shown to induce accumulation of TAMs and secretion of IL-10, TGF-β, and Arginase-1 in oral squamous cell carcinoma and promote an immunosuppressive microenvironment by suppressing T-cell proliferation (Takahashi *et al*, 2017). TAM-derived cytokines also play an important role in immunosuppression. For instance, TFG-β was shown to inhibit the anti-tumour activity of CD8+ T cells by downregulating the expression of cytolytic genes (Thomas and Massague, 2005). Moreover, IL-10 which is expressed by TAMs, CD8^+^ T cells, and tumour cells is an important cytokine in the tumour microenvironment and plays anti-inflammatory, immunosuppressive role that favours tumour escape from immune surveillance. TAM-derived IL-10 suppresses the expression of IL-12, which is considered as a potential anti-tumour cytokine (Matsuda *et al*, 1994).

## TAMs as therapeutic targets

Several reports have provided strong evidence that TAMs are crucial components of the tumour microenvironment and TAM infiltration is strongly associated with poor prognosis and survival rates in cancer patients. Based on these findings, targeting TAMs is emerging as an attractive strategy for therapeutic intervention (Chanmee *et al*, 2014; Yang and Zhang, 2017).

It is now well-established that chemoattractants in tumour microenvironment facilitate massive infiltration of macrophages in tumours. Hence, depletion or inhibition of TAM recruitment by modulating levels of these chemoattractants may serve as an effective strategy. Indeed, pharmacological inhibition, neutralising monoclonal antibodies, or genetic mutation of chemoattractants such as CCL2, VEGFR2, CSF-1R depleted TAM infiltration and reduced tumour growth. For instance, in case of CSF1R, the humanised monoclonal antibody RG7155 potently inhibited CSF1R dimerisation and also induced a striking reduction in the CSF1R+CD163+ macrophage population within tumour tissues (Ries *et al*, 2014). Moreover, PLX3397, a potent tyrosine kinase inhibitor of CSF1R decreased macrophage infiltration thereby enhancing the efficacy of immunotherapy (Mok *et al*, 2014).

Differentiation of pro-tumourigenic M2 to the anti-tumour M1 phenotype is rapidly emerging as a new therapeutic approach. Activation of TLR3/Toll-IL-1 receptor domain-containing adaptor molecule 1 by Poly (I:C) rapidly enhanced secretion of pro-inflammatory cytokines and accelerated M1 macrophage polarisation (Shime *et al*, 2012). Emerging evidence has indicated that abnormalities in tumour vasculature alter the tumour microenvironment and influences tumour progression and responses to cancer therapy. The re-education of TAMs within the tumour could restore normal vasculature and block the pro-tumourigenic effects of TAMs. Indeed, polarisation from an M2 to M1 phenotype suppressed mammary tumour growth and angiogenesis *in vivo* (Zhang *et al*, 2013). According to another report, histidine-rich glycoprotein inhibited tumour growth and metastasis by inducing macrophage polarisation and vessel normalisation via downregulation of the placental growth factor (Rolny *et al*, 2011).

## Concluding remarks

The conventional binary model of macrophage polarisation is becoming antiquated and a newer model of polarisation spectrum is coming into conception, which focuses on involvement of an array of differentiated macrophages in various immunoregulatory disorders as well as cancers. Moreover, it is now well-established that apart from tumour cell-derived factors, stromal microenvironmental factors play a substantial role in tumourigenesis and TAMs constitute as key players in this phenomenon by regulating various steps of tumour initiation, progression, and metastasis. Since the abrogation of immunosuppressive macrophages in the tumour microenvironment enhances anti-tumour response, targeting TAMs is rapidly emerging as a promising therapeutic strategy for cancer patients.
